# Dietary Folic Acid Alters Metabolism of Multiple Vitamins in a CerS6- and Sex-Dependent Manner

**DOI:** 10.3389/fnut.2021.758403

**Published:** 2021-11-05

**Authors:** Keri Barron, Besim Ogretmen, Natalia Krupenko

**Affiliations:** ^1^Department of Nutrition, Nutrition Research Institute, The University of North Carolina at Chapel Hill, Kannapolis, NC, United States; ^2^Department of Biochemistry & Molecular Biology, Hollings Cancer Center, Medical University of South Carolina, Charleston, SC, United States; ^3^Department of Nutrition, The University of North Carolina at Chapel Hill, Chapel Hill, NC, United States

**Keywords:** dietary folic acid, vitamins, metabolism, nutrients interactions, ceramide synthase 6, sex differences

## Abstract

Folic acid, an oxidized synthetic pro-vitamin B_9_, is widely used in vitamin supplement formulations and food fortification to maintain optimal folate status in humans. Studies on folic acid (FA) efficiency in improving folate status and correcting folate deficiency pathologies are abundant, but precise knowledge of FA effects on human and animal tissues is not available. In our recent study, 10-week-old wild-type and CerS6 knockout (KO) mice were placed on FA-deficient, control, or FA over-supplemented diet for 4 weeks. Untargeted metabolomics characterization of mouse liver, brain, and testes tissues after the dietary treatment revealed profound effects of FA on the liver metabolome. Here, we present the analysis of dietary FA effects on tissue concentrations of other vitamins in mice. Despite the expectation that identical dietary supply of the vitamins (excluding FA) to each group should support similar tissue vitamins concentrations, metabolomics data demonstrate significant alterations of tissue concentrations of multiple vitamins by different levels of FA supplementation that were sex- and genotype-dependent. Moreover, we found significant differences in the liver concentration of retinol, thiamin diphosphate, pantetheine, pyridoxal, and pyridoxamine between males and females. While the liver had more changes in vitamins and vitamin derivative levels, the brain tissue and testes also showed changes linked to FA supplementation. Over-supplementation with FA had negative effects on concentrations of vitamins A, B_1_, B_2_, and B_6_, or their metabolites in the liver, but increased intermediates in coenzyme A (CoA) biosynthesis, as well as gamma/beta-tocopherol and phosphorylated forms of B_6_ in the CerS6 KO brain. Overall, our data demonstrate that dietary FA supplementation significantly affects the metabolism of other vitamins, and that these effects depend on the CerS6 status and sex of the animal. Further research is required to determine whether the observed effects are specific to FA, and the mechanisms that are involved.

## Introduction

Folate, an essential nutrient found in natural food sources in a reduced form, is present in vitamin supplements and fortified foods as oxidized synthetic compound folic acid (FA) (1). Despite the wide use of FA to ensure adequate folate status, its effects on biological systems have not been fully elucidated, and significant knowledge gaps related to health effects of excess folate and/or FA have been identified and reviewed (2, 3).

Folic acid has become a part of the American diet since 1998 when fortification of grain products with FA was introduced with the goal of improving population folate status to decrease the incidence of neural tube defects (NTDs) (1). Fortification with FA was very successful in reducing the incidence of neural tube defects in multiple countries (4). However, concerns have been raised with regard to the possibility of unwanted effects of FA and its effect on natural forms of vitamins (1, 5). Folate is a general term for a family of molecules related to FA that participate as cofactors in numerous reactions of one-carbon transfer involved in nucleotide biosynthesis, methylation reactions, and amino acid metabolism (1). Only tetrahydrofolate (THF, fully reduced form) can accept one-carbon units that can then be oxidized or reduced, and donated in biosynthetic reactions to make new molecules (3, 6, 7). Thus, FA absorbed from the diet cannot be used as a cofactor until it is reduced in two sequential reactions and catalyzed by dihydrofolate reductase (DHFR) using nicotinamide adenine dinucleotide phosphate, reduced (NADPH) as a reductant to produce THF. This process starts in the enterocytes, but if supplementation exceeds their capacity, unmetabolized FA will be transported to and metabolized in the liver, which is considered to be the main organ of folate metabolism (8, 9). Initially, it was shown that ingestion of doses ≥200 μg FA resulted in detectable unmetabolized FA in serum (10), but with modern HPLC MS/MS techniques, concentrations of FA above 1 nM were found in over 95% NHANES (National Health and Nutrition Examination Survey, United States) samples (11). The effects of unmetabolized FA have not been fully investigated. Given that, at current levels of dietary folate supplementation, several hundred thousand people are exposed to extra FA for each NTD prevented, the concerns have been voiced with regard to the effects of supplementation with FA (5, 12–14). Thus, a lack of understanding of the effects of FA and folate at various doses, or their mechanisms of action are major gaps that limit the safety and efficacy of supplementation with FA (2, 12–14). Importantly, our early studies found that FA deficiency increased CerS6-dependent ceramide elevation in cultured cells (15), and our recent study has shown that effects of dietary FA in mice are mediated, in part, by ceramide and ceramide-generating enzyme CerS6 (16).

Ceramides, a group of bioactive sphingolipids consisting of sphingoid base linked to an acyl chain *via* an amide bond, are incorporated in cellular membranes as structural components, serve as precursors for complex sphingolipids (sphingomyelins, hexosyl-ceramides, gangliosides), and regulate multiple cellular processes such as cell differentiation, proliferation, survival, and senescence, and response to stress (17–19). Ceramides can be generated from *de novo* biosynthesis of dihydrosphingosine, or *via* the salvage pathway, which includes the degradation of pre-formed complex sphingolipids to sphingosine (18). Six ceramide synthases (CerS1–6) attach acyl chains to dihydrosphingosine or sphingosine, and each isoform has a different preference for the specific length of acyl-CoA chains used to make ceramides (20–23). This results in a specific balance of ceramide species in cells and tissues, which defines the outcome (23, 24). Importantly, specific ceramides have been implicated in the development and progression of cardiovascular disease (25), metabolic syndrome (26), and alcoholic and non-alcoholic liver diseases (27–30). Additionally, ceramides were shown to respond to nutrient stress, including magnesium withdrawal (31, 32), treatment with a synthetic retinoid (33), or vitamin E metabolite γ-tocotrienol (34), and increased supply of fatty acids (35). The elevation of ceramides in response to the aforementioned nutrients occurs *via* the upregulation of *de novo* ceramide biosynthesis as indicated by increased dihydroceramide species (33–35) or through the modulation of the activity of enzymes involved in the metabolism of complex sphingolipids (31).

Ceramide synthase 6, the enzyme generating C_14_- and C_16_-ceramides, is expressed in most tissues at low levels but can be induced in response to stress stimuli (15, 36, 37). Its product, C_16_-ceramide, functions as a lipotoxic mediator of metabolic stress based on a plethora of evidence indicating the detrimental role it plays in both cells and rodent models (38). For this reason, suppression of C_16_-ceramide production is often viewed as a strategy for overcoming the negative impact of stressors. Of note, our studies on cultured cells have shown that impairment of folate-dependent reactions by limiting FA supplementation, as well as by inhibiting or knocking out folate-dependent enzymes, activates ceramide signaling in cultured cells (15, 39). Thus, our study on FA effects was designed to include both WT and CerS6 KO mice in order to identify changes driven by ceramide response to metabolic stress. We observed that both FA and CerS6 knockout affected multiple metabolic pathways (16). Here, we present an untargeted metabolomics evaluation of multiple vitamins and their cofactor concentrations in tissues of mice maintained for 4 weeks on diets with different levels of FA supplementation.

## Materials and Methods

### Animals and Diets

All animal experiments were reviewed and approved by the Institutional Animal Care and Use Committee (IACUC) at the North Carolina Research Campus (NCRC, protocol # 18-011) and accomplished in compliance with the ethical guidelines for the use of animals in research (40). CerS6 knockout (KO, C57Bl/6N background) mice were obtained from the laboratory of Dr. Ogretmen (41), and we bred them back five to six generations to C57Bl/6NHsd mice purchased from Envigo (Indianapolis, IN, United States) to ensure the absence of genetic drift in the experimental animals. Animals for dietary experiments were generated by breeding heterozygous pairs. Offspring from these pairs showed a sex distribution of 50/50 and were genotyped using tail-lysates for PCR with specific primers as described elsewhere (16). Wild-type (WT, CerS6^+/+^, control) and knockout (CerS6^−/−^) littermates, both male and female, were randomized to dietary regimens, with each regimen having four groups, each including five animals per group: WT males, KO males, WT females, and KO females. A power analysis indicated that with five animals per group and an estimated SD of 25%, we should be able to detect 40% differences between the groups with α <0.05 and 80% power. The mice were group-housed in microisolator cages under standard conditions (12 h light/dark cycle, temperature, and humidity control) with *ad libitum* access to water and one of the three purified synthetic diets purchased from Envigo (formerly Teklad). The diets had identical energy densities of 3.8 kcal/g (15.899 kJ/g) with 14.4% kcal/kJ from fat, 66.5% kcal/kJ from carbohydrates, and 19.1% kcal/kJ from protein, and differed only in the amount of folic acid (FA): (1) folic acid-deficient diet (FD, catalog number TD.95247) with no added FA and containing only residual 0.2 ppm FA coming from added protein; (2) control (Ctrl) diet with 2 ppm FA added (TD.160824), and (3) FA over-supplemented (FS) diet containing 12 ppm added FA (TD.160825). The amino acid sources (casein and L-cystine) were consistent across the diets, as were the sources of carbohydrates (corn starch, sucrose, maltodextrin), fat (soybean oil), and all vitamins except FA. Comparative diet composition is presented in Supplementary Table 1. We did not measure FA in the diets because we have over 10 years of experience with this commercial provider and at all times, the observed responses of animal blood folate concentrations were consistent with the supplementation level. Wild-type and CerS6KO littermates, both male and female, were placed on respective diets at 10 weeks of age and maintained on the diets for 4 weeks. At the end of dietary exposure (14 weeks of age), the mice fasted for 4 h before euthanasia. The fasting period before tissue collection was used to synchronize the last ingestion of food in all the groups, which should decrease the variability of tissue vitamin concentrations. Immediately after the animals were euthanized, blood and major tissues were isolated and sampled for histological evaluation and metabolomics analysis. The tail portion of the hepatic left medial lobe was fixed in buffered formalin, while the middle portion of the lobe was aliquoted in several ~100 mg sections, which were snap-frozen in liquid nitrogen for down-stream analysis. Similarly, the left half of the brain and the left testicle from each male mouse were snap-frozen in liquid nitrogen. After this, the snap-frozen tissues and all collected tissue samples were stored at −80°C.

### Untargeted Metabolomics Analysis

The untargeted metabolomic analysis was performed by the commercial service provider Metabolon® (Durham, NC, United States). The snap-frozen liver, brain, and testes tissues were cut, weighed, and shipped to Metabolon® on dry ice. Each sample received by Metabolon was assigned a unique identifier by the LIMS (Laboratory Information Management System) and stored at −80°C. Before the extraction process, several recovery standards were added to each sample for QC purposes. The samples were prepared using a MicroLabSTAR® system (Hamilton, Reno, NV, USA). Precipitation of the proteins, dissociation of the small molecules bound to them or trapped in the protein matrix, and recovery of chemically diverse metabolites were achieved by mixing with methanol under vigorous shaking for 2 min (Glen Mills Geno Grinder 2000) with subsequent centrifugation. The extracts obtained were divided into five aliquots for further analysis by ultrahigh performance liquid chromatography/mass spectrometry (UHPLC/MS) (16, 42, 43). The sample aliquots were briefly concentrated on a TurboVap® (Zymark, Marshall Scientific, Hampton, NH, USA) and stored under nitrogen before preparation for analysis. The global biochemical profiling consisted of four unique arms covering the identification of both hydrophilic and hydrophobic compounds under both positive and negative ionization conditions (43), with one aliquot stored as a backup. Chromatographic conditions for each of the four arms were as described previously (16). Metabolite identification was accomplished by automated comparison of ion features in the samples to a reference library of chemical standards characteristics, which included retention time, m/z (mass/charge) ratio, predominant adducts, and in-source fragmentation and associated spectra. Measurements on 736 identified metabolites were provided, and we have previously reported on findings from lipid families (16). In this study, analysis is focused only on vitamin concentrations in the liver, brain, and testes. The liver has been selected as the main organ of folate metabolism (9) and the main organ handling the nutrients absorbed from the diet. The brain was selected as the organ highly sensitive to the status of many vitamins. Additionally, testes were investigated because our early experiments indicated degenerative changes of these organs in aged CerS6^−/−^ mice. For graphical presentation of tissue vitamin concentrations (boxplots), each biochemical in the original scale was rescaled to set the median equal to 1.

### Statistical Analysis

The statistical analysis of differences between three or more groups was performed by one-way ANOVA. The statistical analysis of the fixed effects of sex, genotype, or diet, as well as their pair-wise interactions and the interaction of all three factors, was completed by Metabolon® by ANOVA comparisons (detailed description presented in Supplementary Material—Metabolon Statistical Analysis). Complete tabulated data of statistical measures are presented in Supplementary Table 2 (columns AR–EA). Differences with the *p*-value < 0.05 were considered significant.

## Results

In our experiments, the mice were fed with purified diets, which differed only in the concentration of FA added to the diet, while concentrations of other nutrients, such as all water- and fat-soluble vitamins and microelements, were identical. However, after 4 weeks on respective diets, the groups demonstrated differences in tissue concentrations of multiple vitamins and related cofactors. In certain cases, the differences were sex- and genotype-dependent.

### Folic Acid Modulated Liver Concentrations of Fat-Soluble Vitamins A and E

#### Vitamin A

**Figure 1:**
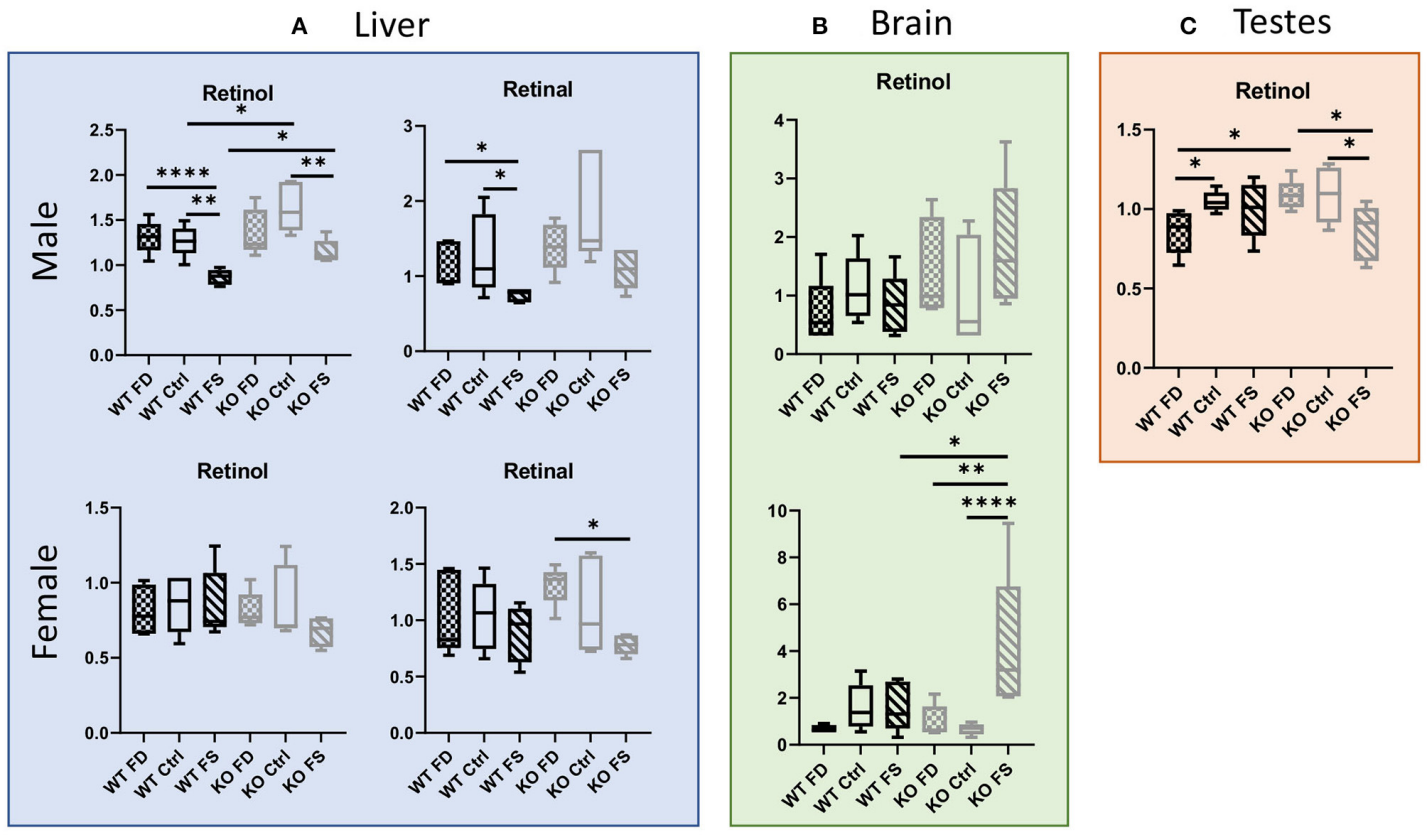
**(A)** Hepatic vitamin A metabolites are altered by dietary folic acid (FA), with changes in females being CerS6-dependent. **(B)** Higher variability of retinol concentrations was found in brains. **(C)** Response of retinol concentrations in testes was also CerS6-dependent. Data show minimum and maximum, with the bar at median value, *n* = 5. Checked bars, folic acid-deficient (FD) diet; solid bars, control (Ctrl) diet; striped bars, FA over-supplemented (FS) diet. Wild-type (WT) is shown in black, and knockout (KO) is shown in gray. *p < 0.05; **p < 0.01; ****p < 0.0001; determined by analysis of variance (ANOVA) comparisons.

The folic acid-over-supplemented (FS) diet, compared with the control (Ctrl) diet, resulted in significantly lower retinol concentrations in livers of both the WT and CerS6 KO male mice (32 and 30%, respectively) (Figure 1A; Supplementary Table 2). Interestingly, retinol concentrations in the CerS6 KO livers were significantly higher than in the WT livers on either the Ctrl or FS diet (29 and 32%), while no difference was observed between the male WT and CerS6 KO mice on the FD diet. Similar to retinol, retinal concentrations in the male livers decreased with increase in dietary FA consumption, but the effect was only significant in the WT mice (0.58- to 0.66-fold change). In the female mice, no significant differences in retinol concentrations between the WT and CerS6 KO mice was observed, but there was a non-significant trend for decreased retinol in the KO FS mice compared with the KO FD and KO Ctrl mice. Notably, retinol concentrations in the female livers were significantly lower than in the male livers (0.53- to 0.67-fold). Retinal concentrations showed few differences in the female WT mice, but a statistically significant difference between the FS and FD groups was found among the KO females (38% decrease).

Retinol concentrations were also measured in brain and testes (Figures 1B,C; Supplementary Table 2). In the brain tissue, no significant difference in retinol concentrations was found in males, with only a trend for increased levels in KO mice on the FS diet compared with WT FS-fed group (*p* = 0.0501). In the females, the KO FS group similarly had a significantly higher retinol concentration compared with both KO groups (6.24-fold higher than the KO Ctrl mice and 4.19-fold higher than the KO FD mice) and with the WT groups. In the testes, retinol was significantly lower in the FS diet-fed KO mice compared with either the Ctrl- or FD-fed KO mice (21–22% lower). This effect of lower retinol concentrations upon FS diet consumption was not seen in the WT mice. Conversely, testes of the WT FD mice had significantly lower retinol levels compared with the WT Ctrl and KO FD mice (0.82-fold and 0.78-fold, respectively).

#### Vitamin E

**Figure 2:**
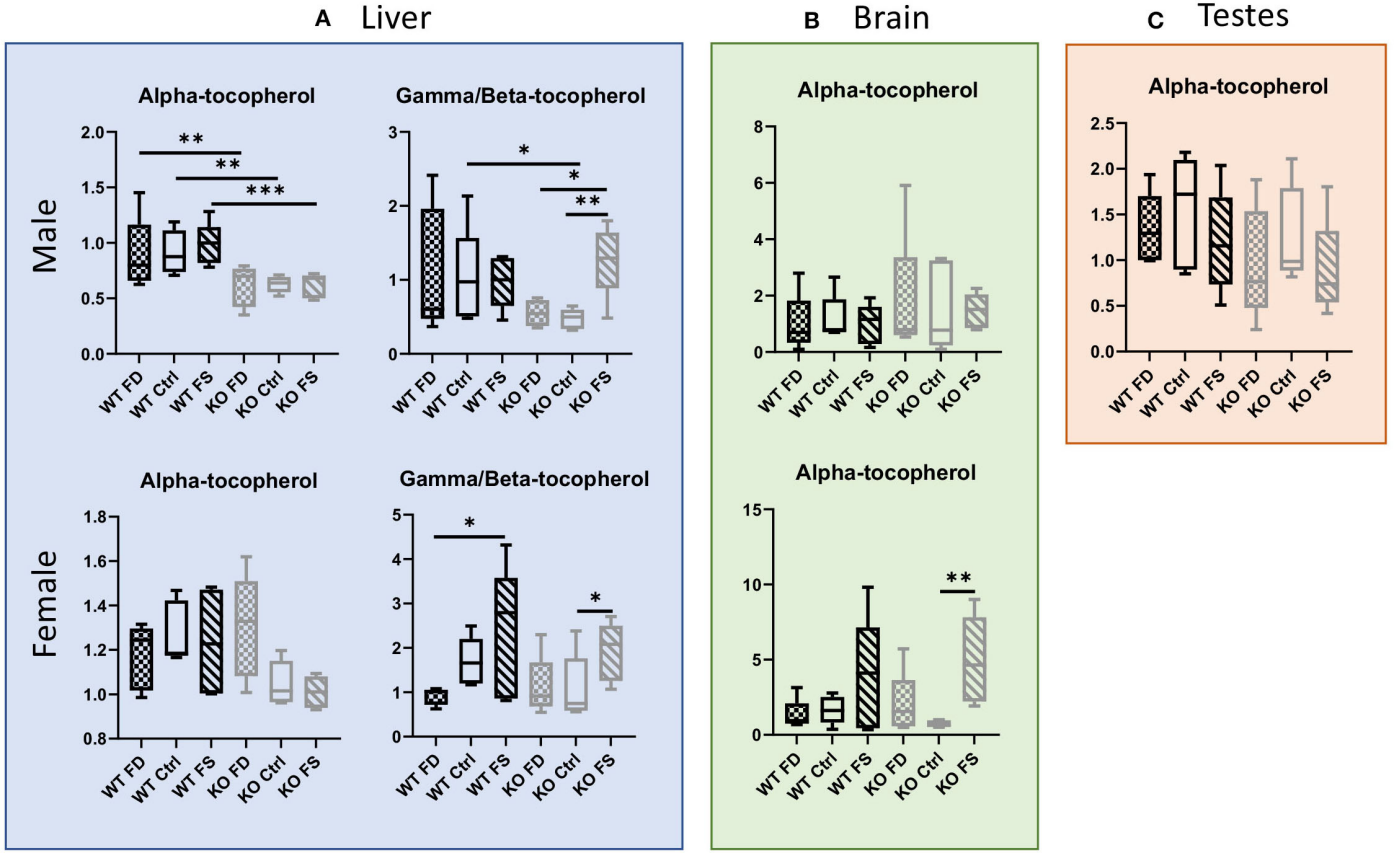
Loss of CerS6 in males resulted in significantly lower concentrations of alpha-tocopherol and a trend for lower gamma/beta-tocopherol, which was rescued by **(A)** FS diet. No significant differences between groups were seen in testes **(C)**. Female liver **(A)** and brain **(B)** showed increase in gamma/beta-tocopherol with FS diet. Data show minimum and maximum, with bar at median value, *n* = 5. Checked bars, FD diet; solid bars, Ctrl diet; striped bars, FS diet. WT is shown in black, and KO is shown in gray. *p < 0.05; **p < 0.01; ***p < 0.001; determined by ANOVA comparisons.

Another fat-soluble vitamin affected by dietary FA was vitamin E. In the liver, alpha-tocopherol concentrations were significantly lower in the male CerS6 KO mice regardless of dietary FA supplementation (0.69-fold for FD, 0.68-fold for Ctrl, and 0.63-fold for FS). In the females, no effect of the FA on alpha-tocopherol was seen in the WT mice (Figure 2A; Supplementary Table 2). However, there was a trend for reduction of vitamer levels on FS diet in the female KO mice (~30% from FD). Similarly, the concentration of gamma/beta-tocopherol was significantly lower in the male KO mice fed with a FD or Ctrl diet compared with the FS diet, but the excess of dietary folic acid rescued this effect (~2.6-fold increase, Supplementary Table 2), with the KO FS mice having similar levels with the WT FS mice. In the female mice, there were no differences between the WT and CerS6 KO mice, but the FS diet increased gamma/beta-vitamer levels in both genotypes (2.6-fold in WT compared with the FD group and 1.8-fold in KO compared with the Ctrl fed KO mice). In male brain tissues, alpha-tocopherol levels showed high variability in both genotypes and between dietary groups, thus no significant differences were found (Figure 2B; Supplementary Table 2). The female mice also had high variability of vitamer concentrations but showed a clear trend for increased alpha-tocopherol in the WT mice on FS diet, as well as a statistically significant increase in the KO mice on FS compared with the Ctrl diet (2.5- and 6.8-fold correspondingly). No significant differences in alpha-tocopherol levels were found between the groups in the testes (Figure 2C; Supplementary Table 2).

### Multiple Water-Soluble Vitamins Were Affected by Dietary FA Supplementation

#### Vitamin B_1_

**Figure 3:**
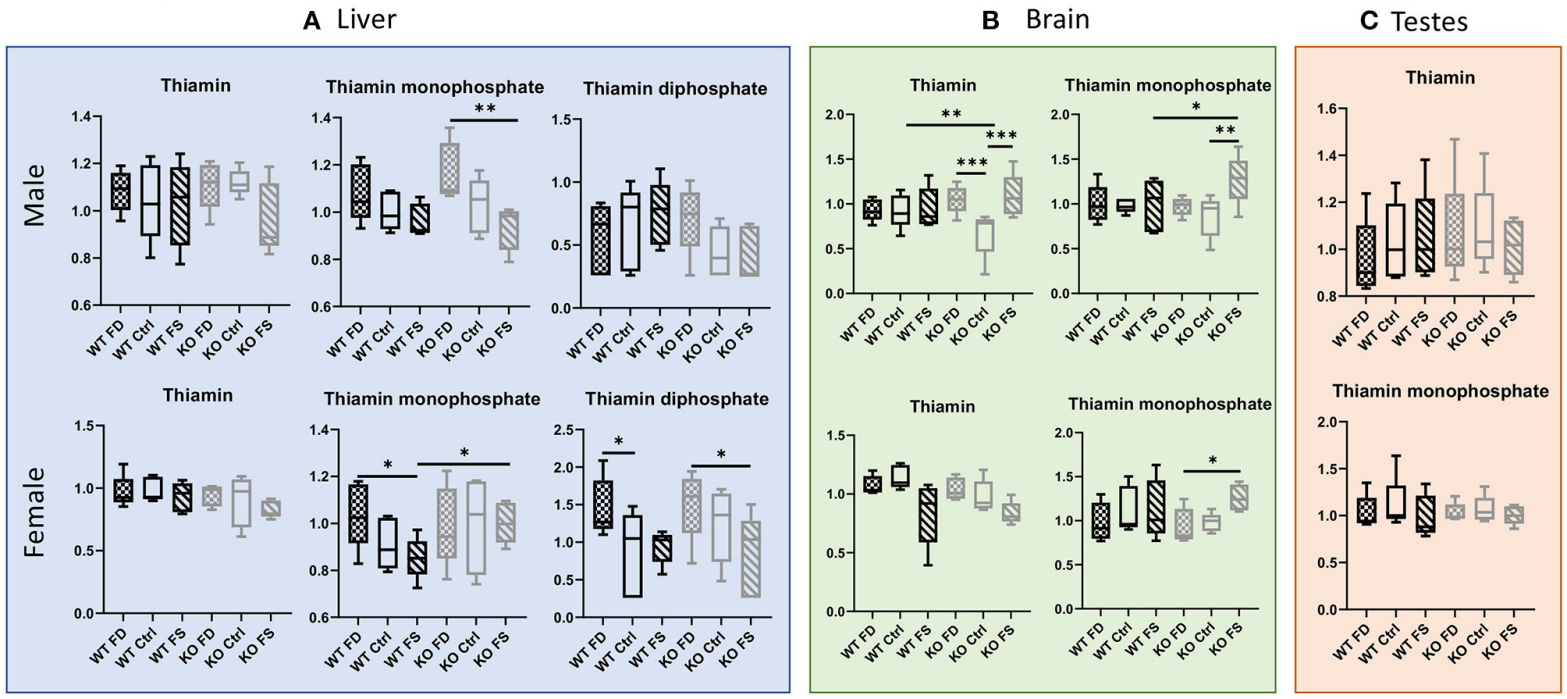
Thiamin concentrations did not differ in livers of both sexes, while the phosphorylated forms were affected by dietary FA **(A)**. FA over-supplementation increased both thiamin and thiamin monophosphate in brains of KO males **(B)**, and no effects on thiamin metabolites were found in testes **(C)**. Data show minimum and maximum, with bar at median value, *n* = 5. Checked bars, FD diet; solid bars, Ctrl diet; striped bars, FS diet. WT is shown in black, and KO is shown in gray. *p < 0.05; **p < 0.01; ***p < 0.001; determined by ANOVA comparisons.

While liver concentrations of vitamin B_1_ (thiamin) itself were not significantly different among all the experimental groups (Figure 3A; Supplementary Table 2), the phosphorylated forms of the vitamin showed differences between the groups with different dietary FA supplementation. Thiamin monophosphate demonstrated a negative association with dietary FA in the male mice as well as in the female WT mice. The difference was significant between the FD- and FS-fed mice for male CerS6 KO (20% decrease) and female WT mice (18% decrease). The female KO mice did not show the same pattern for thiamin monophosphate concentrations in liver. Thiamin diphosphate concentrations (TPP, active cofactor) were higher in female than in male livers (by a factor of 1.99–2.77 in the KO mice and by a factor of 1.25–2.6 in the WT mice, Supplementary Table 2). Moreover, TPP concentrations showed a modest increase with increase in FA in the diet in WT males only, although the changes did not reach statistical significance. Surprisingly, TPP concentrations in the KO males and both the WT and KO females decreased with increase in dietary FA and reached statistical significance when the FS groups were compared with the FD in female KO mice (45% decrease, Figure 3A; Supplementary Table 2). No significant changes depending on FA supplementation were seen in the WT males, while the KO males demonstrated a trend for cofactor reduction.

Similar to liver, brain concentrations of thiamin showed no differences between diets both in the WT males and females (Figure 3B; Supplementary Table 2). In the female KO mice, there was a trend of reduced B_1_ concentrations with increase in dietary FA supplementation, while the KO males showed a U-shaped relationship with dietary FA, which was statistically significant. For thiamin monophosphate, FA supplementation had no effect on brain concentrations in WT males or females, but the CerS6 KO mice of both sexes responded with a statistically significant increase at the highest FA supplementation compared with the lowest FA supplementation (31 and 35% increase for KO males and females, correspondingly, Figure 3B; Supplementary Table 2). There were no significant differences between the dietary groups in thiamin or thiamin monophosphate concentrations in testes (Figure 3C; Supplementary Table 2).

#### Vitamin B_2_

**Figure 4:**
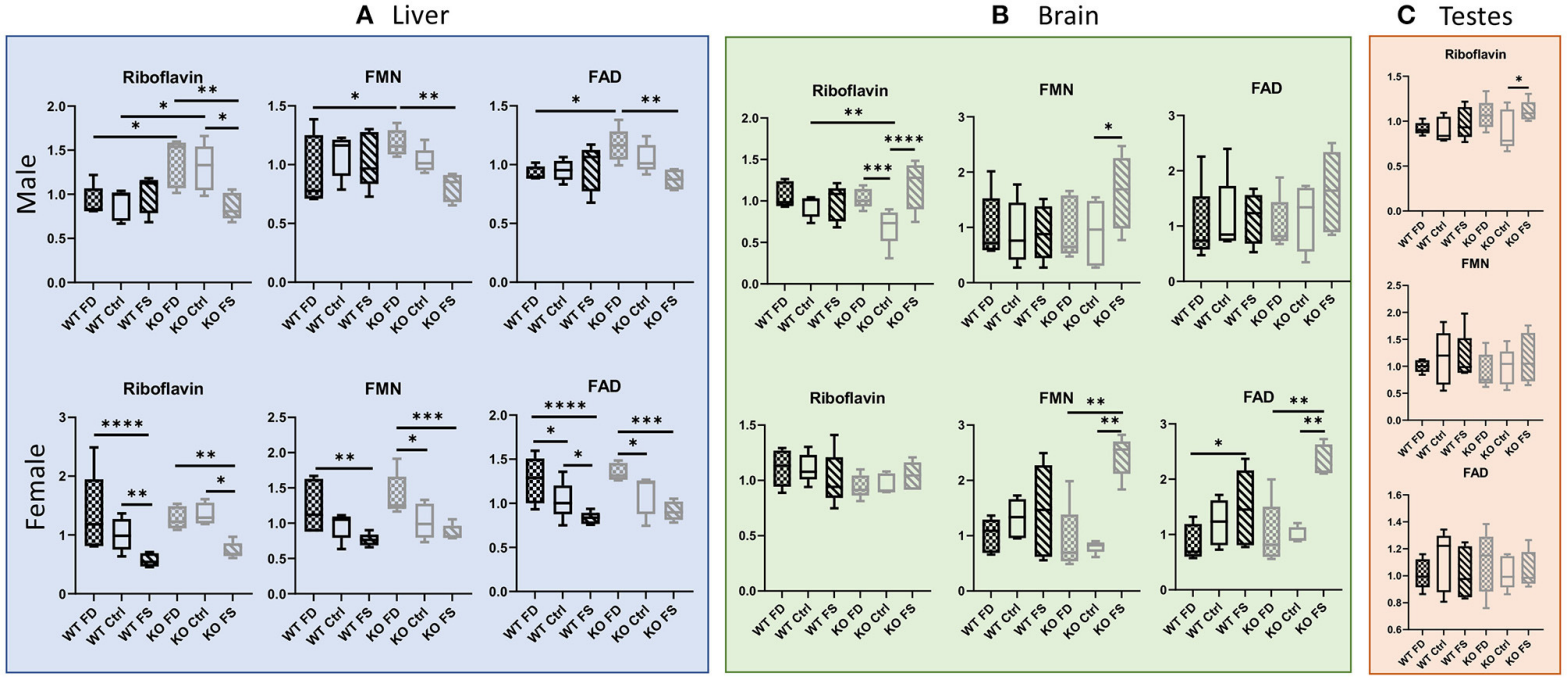
High dietary FA decreased liver concentrations of riboflavin, FMN and FAD in females of both genotypes and KO males, but not in WT male livers **(A)**, brains **(B)**, or testes **(C)**. Female brains showed no difference for riboflavin in both genotypes **(B)**. Trend for increased FMN and FAD on FS diet was found for KO brains in both sexes **(B)**. Data show minimum and maximum, with bar at median value, *n* = 5. Checked bars, FD diet; solid bars, Ctrl diet; striped bars, FS diet. WT is shown in black, and KO is shown in gray. *p < 0.05; **p < 0.01; ***p < 0.001; ****p < 0.0001; determined by ANOVA comparisons.

Vitamin B_2_, riboflavin, demonstrated a statistically significant negative association with dietary FA levels in the female (WT and KO) as well as in the KO male livers. Interestingly, no differences in liver concentrations of riboflavin were found in the WT males at any level of FA supplementation despite being lower in concentration than in the KO male mice on the FD and Ctrl diets (Figure 4A; Supplementary Table 2). In the females, there were no significant differences in B_2_ between the WT and KO mice, and the effects of dietary FA were similar, with both FS diet groups having significantly lower levels of riboflavin compared with the FD and Ctrl groups (43–62% reduction, Supplementary Table 2). The cofactor forms Flavin Mononucleotide (FMN) and Flavin Adenine Dinucleotide (FAD) in the mouse livers exhibited a similar pattern of response: The WT male livers were mostly insensitive to dietary FA, whereas the KO male livers and both the WT and CerS6 KO female livers demonstrated an inverse relationship with dietary FA levels.

In the brain tissue, the WT male mice exhibited no differences in riboflavin concentrations, whereas the CerS6 KO mice exhibited a U-shaped response, with riboflavin concentrations measuring significantly lower in the Ctrl diet fed mice compared with the FD- and FS-fed mice (68 and 59%, correspondingly, Figure 4B; Supplementary Table 2). The concentration in the brain tissue of the KO-Ctrl mice was also significantly lower compared with WT-Ctrl concentrations (26%). However, no significant differences were found in the brains of the female mice. The male mice displayed high variability in both FMN and FAD concentrations, and only the CerS6 KO brains showed a trend for increasing mean cofactor concentrations with increase in dietary FA, which is opposite to the effect observed in the liver. In the female mice, both WT and CerS6 KO, brain concentrations of FMN and FAD demonstrated significant increases at highest level of FA supplementation. For FMN, this trend was not significant in the WT brains, but in the KO brains, the FS-fed mice had significantly higher concentrations (2.69- to 3.05-fold) compared with the FD and Ctrl-fed mice. The KO FS mice had a 2.32- to 2.35-fold increase in FAD concentration compared with KO FD and KO Ctrl, respectively, and the WT FS mice had a 1.92-fold increase compared with the WT FD mice.

Riboflavin concentrations in testes (Figure 4C; Supplementary Table 2) varied less than in liver or brain with one significant difference where riboflavin concentration increased by 24% in the KO FS mice compared with KO Ctrl. No differences between the groups were found in FMN or FAD concentrations.

#### Vitamin B_3_

Levels of both B_3_ vitamers, nicotinamide, and nicotinic acid (nicotinate), showed sex- and genotype-dependent response to dietary FA; however, differences in vitamer concentrations often did not reach significance (Supplementary Figure 1; Supplementary Table 2). In the liver tissue of the male and female mice, the highest dietary FA supplementation resulted in the reduction of concentration of several vitamers for the WT animals. The concentrations of nicotinamide and nicotinate in the female KO mouse livers did not respond to FA supplementation. Nicotinamide and nicotinate ribosides followed a trend of being lower in FA-supplemented mice in both and females, while the nucleotide forms of the vitamin (NMN and NAD) showed no significant differences at different FA supplementation in males but trended to be lower (~20%) for NAD^+^ in the over-supplemented females of both genotypes. The degradation products 1-methylnicotinamide and nicotinamide N-oxide did not show significant differences at different levels of FA supplementation; however, the latter was significantly higher in the female livers than in the male livers (1.88-fold for KO Ctrl and 1.99 for WT FD). Another metabolite derived from NAD, ADP-ribose, which is the degradation product of poly-ADP-ribosylation or of the cADP-ribose hydrolysis by CD38, was increased with increase in dietary FA in the WT mice of both sexes and in the KO males, but not in the KO females. Of note, WT liver concentrations of ADP-ribose in females on the FD and FS diets were 4.86 and 1.88 times higher than in the WT males and in the KO females, and 15.11 and 2.18 times higher than in the KO males on corresponding diets. Ctrl diet WT females had 1.85-fold higher ADP-ribose concentrations than males with the difference between KO females and males being negligible (Supplementary Table 2).

In the male mice brain tissues, no significant differences were found in vitamers or related B_3_ metabolite concentrations for different FA groups in both genotypes, with the exception of NADH, which progressively increased with the dietary FA increase in the WT mice only (Supplementary Figure 2; Supplementary Table 2). At the same time, female brain tissues exposed to higher FA supplementation showed significantly higher concentrations compared with the Ctrl diet-fed mice for nicotinamide riboside (1.75-fold), NMN (2.01-fold), NAD^+^ (4.39-fold) in the KO animals, but not in the WT mice. Similar to male brains, no significant differences in concentrations of vitamers or metabolites (such as NADH) between different supplementation groups were found in testes (Supplementary Figure 3; Supplementary Table 2).

#### Vitamin B_5_

**Figure 5:**
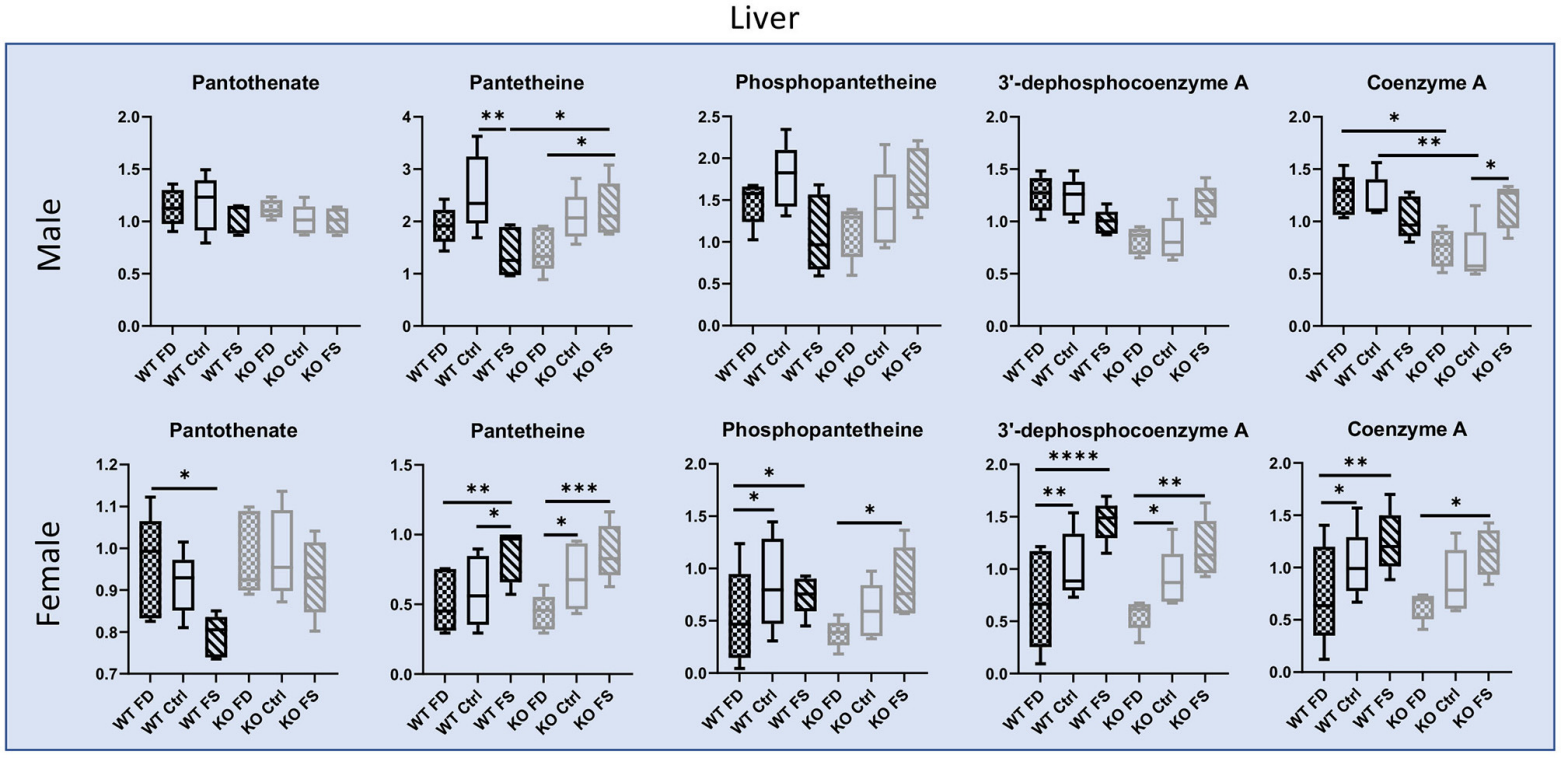
Vitamin B_5_ liver concentrations in males show no difference between genotypes or FA supplementation, while B_5_ metabolites responded to dietary FA differently in WT and CerS6 KO male mice. Most pantothenate metabolites showed a dose response relationship with dietary FA in female mice except pantothenate. Data are shown with minimum and maximum, with bar at median value, *n* = 5. Checked bars, FD diet; solid bars, Ctrl diet; striped bars, FS diet. WT is shown in black, and KO is shown in gray. *p < 0.05; **p < 0.01; ***p < 0.001; ****p < 0.0001; determined by ANOVA comparisons.

In the liver, pantothenate (B_5_) did not differ significantly between males and females for both genotypes and did not respond to dietary FA, with exception of the WT females, which had statistically significantly decreased concentrations of liver pantothenate at highest FA supplementation (17% reduction in WT FS compared with WT FD) (Figure 5; Supplementary Table 2). Concentrations of pantetheine and phosphopantetheine were lower in female than in male livers (39–77% lower), while concentrations of dephosphocoenzyme A and coenzyme A did not show significant differences between sexes. Additionally, all intermediates of coenzyme A biosynthesis (including CoA) in the WT and KO female livers showed significant increases (trends in KO males) with increase in FA supplementation. However, the WT males had a trend for lowest concentrations of these metabolites at the highest FA supplementation (0.84-fold lower) (Figure 5; Supplementary Table 2).

**Figure 6:**
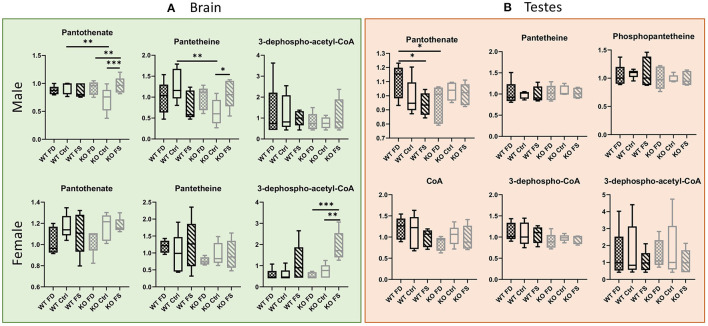
B_5_ metabolites in male **(A)** brain and **(B)** testes show fewer differences between genotypes or dietary FA. In females, most pantothenate metabolites showed FA dose-dependent trend for KO, but not WT mice. Data are shown with minimum and maximum and a bar at median value, *n* = 5. Checked bars, FD diet; solid bars, Ctrl diet; striped bars, FS diet. WT is shown in black, and KO is shown in gray. *p < 0.05; **p < 0.01; ***p < 0.001; determined by ANOVA comparisons.

In the brain, both pantothenate and its metabolites showed no significant differences between males and females (Figure 6A; Supplementary Table 2). Statistically significant increases in pantothenate and pantetheine were seen between Ctrl and FS diets in the KO males (35 and 68% increase in the KO FS males, respectively) and a trend in the KO females, which showed a significant increase in dephospho-acetyl-CoA (3.56-fold increase). Fewer changes were found in testes where pantothenate was significantly reduced by 15% because of the FS diet in the WT mice (Figure 6B; Supplementary Table 2), while most of the other metabolites did not show significant differences.

#### Vitamin B_6_

**Figure 7:**
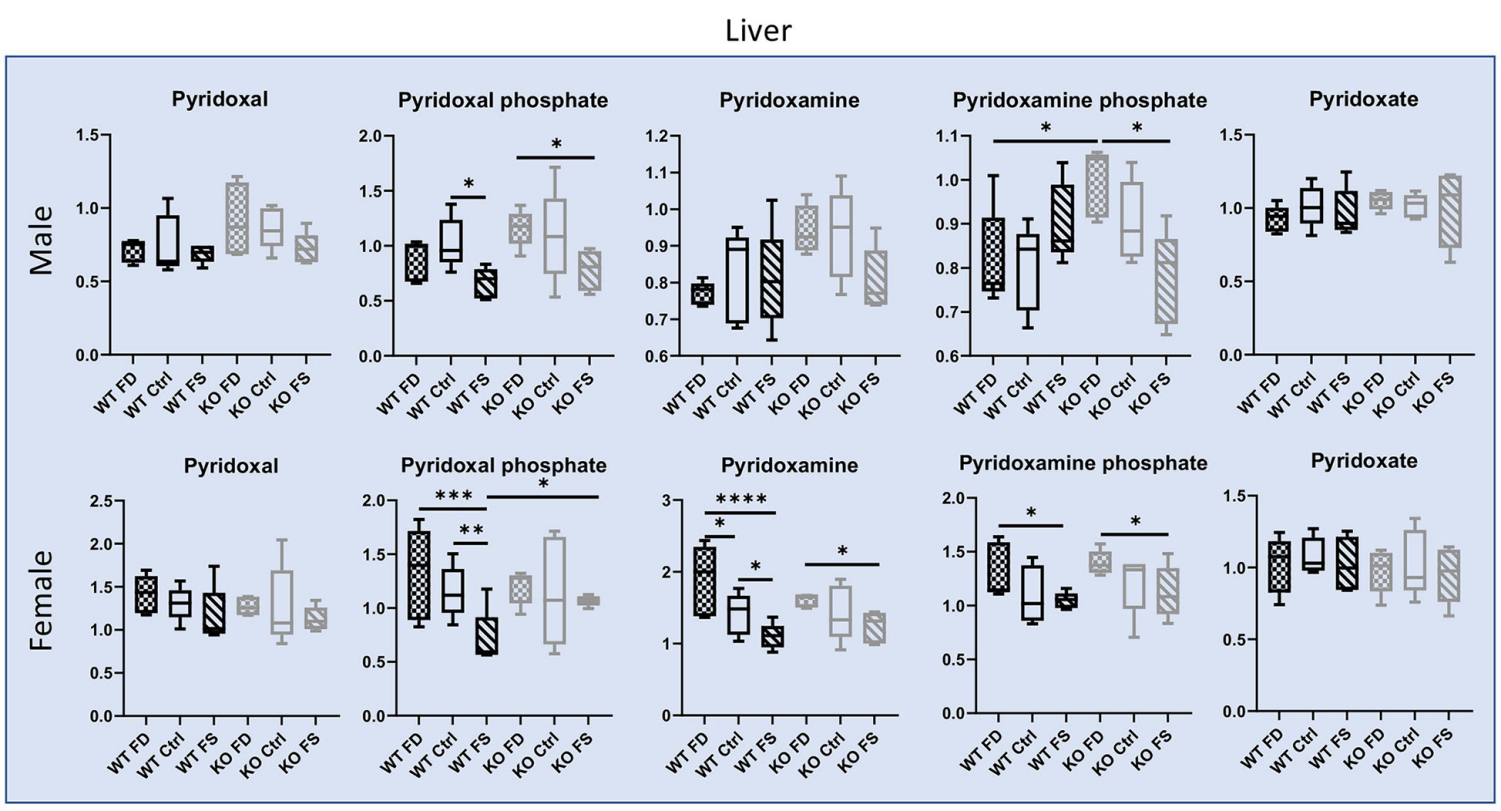
Liver concentrations of B_6_ vitamers and their phosphorylated forms are significantly higher in female vs. male livers, and dietary FA altered several vitamin forms, except the degradation product pyridoxate. Data show minimum and maximum, with bar at median value, *n* = 5. Checked bars, FD diet; solid bars, Ctrl diet; striped bars, FS diet. WT is shown in black, and KO is shown in gray. *p < 0.05; **p < 0.01; ***p < 0.001; ****p < 0.0001; determined by ANOVA comparisons.

With regard to B_6_, the vitamin forms pyridoxal, pyridoxamine, and pyridoxamine phosphate were significantly higher in female vs. the male livers (1.36- to 2.45-fold, Supplementary Table 2), and the female livers showed a trend for these forms to inversely associate with folate supplementation (Figure 7; Supplementary Table 2). In the males, pyridoxamine and pyridoxamine phosphate showed a similar trend, but in the KO mice only. At the same time, concentrations of the cofactor form pyridoxal phosphate were not significantly different between males and females or between genotypes but demonstrated a trend of being lower at highest concentrations of FA. No effects of the dietary FA on the degradation product of B_6_, pyridoxate, were found for both genotypes either in males or females.

**Figure 8:**
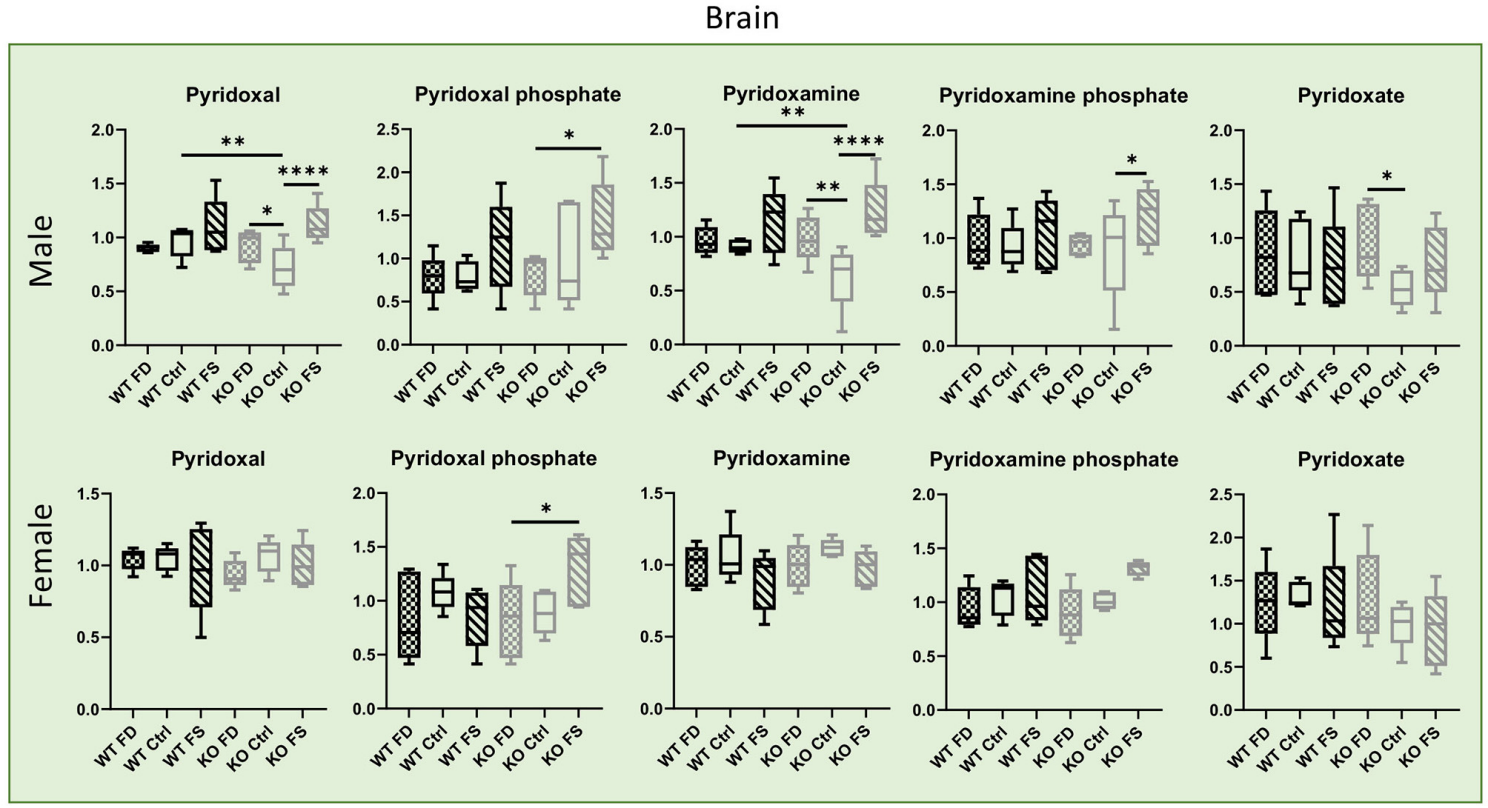
In mouse brains, concentrations of B_6_ vitamers and their phosphorylated forms show no difference between males and females. Significant increase in phosphorylated forms by FA over-supplementation was found in CerS6 KO male brains, but less in female. Data show minimum and maximum with bar at median value, *n* = 5. Checked bars, FD diet; solid bars, Ctrl diet; striped bars, FS diet. WT is shown in black, and KO is shown in gray. *p < 0.05; **p < 0.01; ****p < 0.0001; determined by ANOVA comparisons.

Contrary to the liver, most of the vitamers in the brain tissues showed no differences in concentrations between sexes, with the exception of the degradation product pyridoxate that was significantly higher in females (64–86% higher) (Figure 8; Supplementary Table 2). Both the WT and KO male mice showed a trend for increased concentration of both vitamers and cofactors in the brain with increasing in dietary FA. However, in the female mice, such trend was found for the phosphorylated forms of the vitamin only, and only in the KO mice.

#### Vitamin C

Both ascorbic and dehydroascorbic acid are considered to be vitamin C vitamers, and neither showed changes in the WT male livers upon changing dietary FA (Supplementary Figure 4; Supplementary Table 2). However, in the KO male livers, the concentrations of ascorbate increased with dietary FA increase, while dehydroascorbate decreased with FA increase. Diminishing liver dehydroascorbate with increase in dietary FA was also found in both WT and KO females. Female liver ascorbate levels were reduced with FA increase only in the WT mice, while no changes were found in the KO livers. Threonate, a metabolite linked to vitamin C degradation, was significantly reduced in the FS male livers of both genotypes (21% compared with WT FD and 26% compared with KO Ctrl), but no significant changes were found in the females. Another metabolite, often associated with ascorbate degradation, oxalate, did not show significant effects of FA supplementation either in males or females, but the Ctrl diet KO male livers had significantly higher (43%) oxalate concentrations compared with WT.

In the brain (Supplementary Figure 5; Supplementary Table 2), both vitamer forms were higher in KO than in the WT mice in both sexes, but the differences did not reach statistical significance. The degradation products in males showed no differences between dietary groups, while in females, threonate increased at highest FA supplementation in the WT mice (1.6- to 2.42-fold), while oxalate showed reduction at highest supplementation, also in the WT mice (0.61- to 0.7-fold). The testes showed a tendency for ascorbate increase at highest FA dose, while other metabolites had no significant changes (Supplementary Figure 6; Supplementary Table 2).

## Discussion

In this study, untargeted metabolomics revealed that dietary FA affects the metabolism of multiple vitamins. Since supplementations of all vitamins (excluding FA) were identical in all diets, and these vitamins did not use FA transporters for absorption, initial expectations were that these vitamins and their metabolite/cofactor forms should be at similar concentrations in mouse tissues from different dietary groups. However, untargeted metabolomic data showed a different picture. The tissue concentrations of multiple vitamins or derived cofactors were different between groups, depending on the levels of folate supplementation, sex, and genotype. A closer examination of vitamin concentrations revealed previously unknown responses of both water and fat-soluble vitamins to changes in dietary FA.

Notably, we have recently shown that liver reduced folates themselves demonstrate an unusual response to dietary FA supplementation: FA and 7,8-DHF concentrations progressively increased with increase in dietary FA, while the concentrations of 5M-THF were significantly lower on the FS diet (16). These results concur with a published article on FA over-supplementation (20 ppm) for 6 months in WT and *Mthfr*^+/−^ mice, which found that FA inhibited MTHFR activity *in vitro*, reduced enzyme concentrations in over-supplemented animals, and also increased phosphorylation of the enzyme, which reduced its activity (13). This reduction in 5M-THF on the FS diet is probably limited to the liver only, since peripheral tissues obtain this vitamin from blood, where 5M-THF comprises predominant a fraction of 83–95% (14, 44). Indeed, in the testes, 5M-THF increased proportionately to FA supplementation and was highest on the FS diet (16).

Moreover, the metabolomics data show that in the liver, fat-soluble vitamins A and E concentrations changed with change in dietary FA. Over-supplementation with dietary FA significantly reduced liver retinol and retinal concentrations in the WT and KO male and in the KO female mice but not in the WT females. Overall, retinol concentrations were higher in males than in females. It is not clear why there is a difference in vitamin A level between males and females, or how over-supplementation with FA can lower retinol and retinal concentrations. Increased promoter methylation and reduced expression of RARß2 have been shown upon media FA increase in cultured cells (45), but there are no data available on retinol absorption, transport, or storage, as well as expression of target genes. Potentially, alterations in several lipid classes caused by high FA (16) could affect liver stores of retinol, but this link has not been investigated. Similarly, vitamin E concentrations in the liver and brain were also affected by dietary FA supplementation, and the changes varied depending on genotype and sex. While there are no data on how water-soluble FA could affect tissue concentrations of fat-soluble vitamins A and E, the fact that two moles of NADPH per mole FA are required for incorporation of FA into the folate cycle (1) points to depletion of reductive potential and increase in oxidants that may negatively affect the metabolism of tocopherols. This mechanism, however, cannot explain the increase in vitamin concentrations at higher FA supplementation.

Thiamin liver concentrations were not significantly different between the dietary groups in both male and female mice, indicating that cellular vitamin transport was not affected by FA. However, active coenzyme form TPP concentrations were different depending on dietary FA and animal sex. Sex difference in the concentrations of this cofactor could be linked to the differences in the concentrations of enzymes utilizing it, and while there are no data on enzyme expression in males vs. females, a higher level of pyruvate dehydrogenase complex activity was found in brain mitochondria of young adult female mice compared with young adult male mice (46), which could be a reflection of a higher enzyme expression and consequently lead to higher cofactor concentrations. While the male WT mice did not show changes in TPP, the KO males had decreased concentrations with increase in dietary FA, similar to the females. It is not clear why the active coenzyme would be reduced as dietary FA was increased, but one of the explanations could be the reduced availability of tissue ATP required to phosphorylate the imported vitamin in the cells. At present, there is no information regarding FA over-supplementation effects on hepatocyte ATP concentrations; however, our metabolomics data showed a significant accumulation of succinyl-carnitine in over-supplemented males and females as well as reduced isocitrate concentrations in over-supplemented groups, with the exception of WT females (16). These changes in TCA cycle metabolites could reflect the impairment in cellular ATP production, which merits further investigation. Alternatively, upregulation of the RFC expression by FA could ensue increased export of TMP from the cells (47), thus, reducing the monophosphate form concentration and leading to reduction of the diphosphate coenzyme form. This possibility, though, needs to be tested experimentally.

Our metabolomics measurements revealed significantly higher liver concentrations of both B_6_ vitamin forms and the active cofactor form pyridoxal phosphate in females than in males. At present, the mechanisms causing the differences in liver vitamin concentrations between males and females are not clear: both the transport inside the cells and the efficiency of trapping vitamin in the hepatocytes could contribute to the differences. Importantly, increase in FA supplementation reduced concentrations of pyridoxal, pyridoxamine, and pyridoxamine phosphate in the females and KO males (not always reaching statistical significance), but not in the WT males. These data point to the role of CerS6 and sphingolipids in dietary response. Because the concentrations of the B_6_ degradation product pyridoxate were similar between sexes and did not change with FA supplementation, it is unlikely that differences in tissue concentrations of vitamin forms are caused by vitamin degradation. Pyridoxal phosphate is used as a cofactor by over 150 enzymes (48) and is involved in folate metabolism serving as a cofactor for SHMT, an enzyme which brings one-carbon groups to folate cycle (49). However, it is still not clear how FA could affect tissue concentrations of this cofactor. In contrast to the liver, B_6_ forms in the brain tissues did not differ between males and females but were significantly higher in the FA over-supplemented male CerS6 KO mice. Likewise, few differences between diets were seen in females. Interestingly, pyridoxate concentrations were higher in female brains vs. male, indicating higher B_6_ oxidation in the female brain tissues that could explain the similarity in cofactor and vitamin form concentrations between sexes.

Riboflavin is also connected to folate cycle by being precursor of FAD and FMN, coenzymes for the methylene-tetrahydrofolate reductase [MTHFR (50)] and methionine synthase reductase (51), thus supporting the flow of one-carbon groups to methyl transfer reactions. Our metabolomic analysis shows that increase in dietary FA reduces liver concentrations of the vitamin and its cofactor forms FMN and FAD in the WT and KO female mice and in the KO male mice, which could affect methylation reactions in the liver. Interestingly, an intervention study on the effects of folate and riboflavin supplementation on lowering plasma homocysteine in humans also found that supplementation with FA (400 μg/day) decreased the riboflavin status in humans (52). While increased rate of B_2_ turnover and increased binding of FAD to MTHFR were proposed as possible mechanisms (52), studies on riboflavin interaction with FA and its regulation by FA are missing. Interestingly, in our study, the concentrations of riboflavin, FMN, and FAD in the WT males were not affected by FA supplementation. Moreover, the vitamin concentrations in WT livers from the Ctrl and FD diets were significantly lower than in the KO animals, suggesting that membrane lipid composition is important for nutrient acquisition by the tissue. Indeed, riboflavin is taken by cells *via* multiple complex mechanisms such as specific transporters RFVT1-3 and clathrin-mediated endocytosis [responsible for ~80% of total internalized vitamin in A431 cells (53)], all of which could be affected by membrane lipids (such as ceramides and sphingomyelins). Since the depletion of cellular ATP did not alleviate the intracellular accumulation of riboflavin, the contribution of the passive mechanisms has also been acknowledged. On the other hand, one of the riboflavin family transporters, RFVT2 (SLC52A2), was shown to also mediate efflux of the vitamin (54) and, thus, could potentially reduce tissue concentrations of B_2_.

With regard to niacin, its requirement for mice has been considered controversial because of high efficiency of its synthesis from tryptophan *via* the kynurenine pathway (55). Therefore, the estimated dietary requirement for mice (15 mg of nicotinic acid/kg) is actually based on requirements for rats under the most adverse conditions with regard to tryptophan intake. In our experiments, all the diets had tryptophan and niacin content as recommended, with the expectation that all needs of the animals in corresponding metabolites and cofactors will be met. Indeed, we observed few differences in nucleotide forms of the vitamin, NMN, and NAD^+^, in males, and for the NMN in females between different levels of FA supplementation. However, increasing dietary FA supplementation significantly lowered the liver NAD^+^ in females that could be explained by increased requirement in NADPH for the reduction of FA to THF. It is not clear, however, why this depletion is seen only in the females. Interestingly, liver ADP-ribose concentrations were significantly higher in the female livers than in the male livers and showed a significant response to changes in FA supplementation in the males (both genotypes) and in the WT females. Since ADP-ribose is a highly potent agonist of the TRPM2 calcium channel (56, 57), the FA-related elevation of ADP-ribose could point to the activation of still unidentified signaling pathways in the liver. Interestingly, both the brain and testes showed different patterns of niacin and its metabolites response compared with the liver, apparently, because of exposure to the blood pools of the vitamin that are maintained by the liver. While brain niacin metabolites showed sex-dependent responses to FA supplementation, no significant changes in response to FA were observed in the testes. This distinct vitamin response could be related to the differences in tissues metabolism.

Vitamin B_5_ (pantothenate) did not differ between dietary groups in the male livers but was reduced or showed a trend for reduction in the female livers. Conversely, the concentrations of the cofactor form CoA showed a correlation with dietary FA supplementation. The correlation of CoA changes with FA levels could be linked to the involvement of cysteine and ATP in coenzyme A biosynthesis. Both metabolites were affected by FA supplementation (16). However, the trend for reduction of all intermediates of CoA biosynthesis in the WT males cannot be explained at present.

While ascorbate and dehydroascorbate have been required nutrients for humans and several other species, the lack of its requirement for mice has been established a long time ago. Nevertheless, ascorbate is added to diets to increase animal life span and reproductive function (55). In agreement with the ability to synthetize ascorbate, few differences in the vitamin and its degradation product concentrations were found in liver.

Overall, our data demonstrate perturbations in the metabolism of multiple vitamins upon FA over-supplementation, which could affect numerous metabolic pathways. Mechanisms of these effects are not known; however, both direct and indirect influences of FA may be involved. Significant differences between males and females were observed for vitaminsB_2_, B_5_, and B_6_, pointing to sex differences in vitamin metabolism, a critical aspect that is not studied yet. Importantly, the knockout of CerS6 resulted in a shift from the WT vitamin-FA relationship pattern to the relationship pattern of the opposite sex. For example, for thiamin diphosphate, pyridoxamine phosphate, pyridoxal, CoA, and riboflavin, the pattern of response to FA in the male KO livers was more similar to a female response pattern than to a WT male response. At the same time, for pyridoxal phosphate and retinol, the pattern of response to FA in the KO females was similar to male response patterns and not to a WT female response. While multiple signaling and regulatory roles for C_16_-ceramide have been established, current knowledge of its effects on vitamin metabolism is lacking.

## Conclusion

In conclusion, our study demonstrates that the consumption of low or high dietary folic acid alters the metabolism of other vitamins, with CerS6 status and sex modifying some of these effects. The main strengths of our study are (i) investigation of both low and high FA supplementation in comparison with control supplementation, (ii) employment of the untargeted metabolomics approach, and (iii) evaluation of the effects of dietary FA in both sexes. There is little evidence linking metabolism of different vitamins, and our data underscore the importance of understanding how different vitamins are affecting each other in cellular metabolism. This study has also its limitations, stemming from the fact that it represents a part of a bigger study that was designed with a broader aim of investigating the role of ceramide and CerS6 on the folate stress induced by low or high FA supplementation (16). Since at that stage we were not aware of the changes in other tissue vitamins, the experiments of “rescuing” FA effects by the supplementation of individual vitamins, as well as investigation of the mechanisms of FA effects, were not included in the project. Overall, our experiments reveal that both dietary FA and CerS6 have pleiotropic effects on liver metabolome (16), and that some of these effects, such as alterations in tissue concentrations of other vitamins or their cofactor forms, cannot be linked directly to folate metabolism but could be mediated by additional regulators. Further studies on mechanisms connecting folate and sphingolipid metabolism, as well as characterization of folic acid effects on liver metabolic pathways, will help to develop strategies allowing to avoid the potential unwanted side effects of over-supplementation.

## Data Availability Statement

The original contributions presented in the study are included in the article/Supplementary Material, further inquiries can be directed to the corresponding author.

## Ethics Statement

The animal study was reviewed and approved by IACUC at the North Carolina Research Campus, UNC, Kannapolis, NC. All experiments were conducted in compliance with the ethical guidelines for the use of animals in research.

## Author Contributions

Conceptualization, experimental design, implementation, and data analysis were conducted and the original draft of the manuscript was written and prepared by KB and NK. The CerS6 KO mice were generated by BO. The discussion of results, manuscript review, and editing were performed by KB, NK, and BO. Funding acquisition and project administration were carried out by NK. All authors contributed to the article and approved the submitted version.

## Funding

The study presented in this manuscript was supported by the National Institutes of Health grant CA193782 to NK and BO was supported by CA203628, CA214461, and DE016572.

## Conflict of Interest

The authors declare that the research was conducted in the absence of any commercial or financial relationships that could be construed as a potential conflict of interest.

## Publisher's Note

All claims expressed in this article are solely those of the authors and do not necessarily represent those of their affiliated organizations, or those of the publisher, the editors and the reviewers. Any product that may be evaluated in this article, or claim that may be made by its manufacturer, is not guaranteed or endorsed by the publisher.

## Abbreviations

FA: folic acid
7,8-DHF: 7,8-dihydrofolate
THF: tetrahydrofolate
5M-THF: 5-methyl-tetrahydrofolate
MTHFR: methylene-tetrahydrofolate reductase
FD: folate deficient
Ctrl: control
FS: folate over-supplemented
CerS: ceramide synthase
WT: wild-type
KO: knockout.
