# Supplementary figures and images for: Dietary Folic Acid Alters Metabolism of Multiple Vitamins in a CerS6- and Sex-Dependent Manner
Source: Front Nutr. 2021 Nov 5;8:758403. doi: 10.3389/fnut.2021.758403 (PMC8602897; doi:10.3389/fnut.2021.758403)

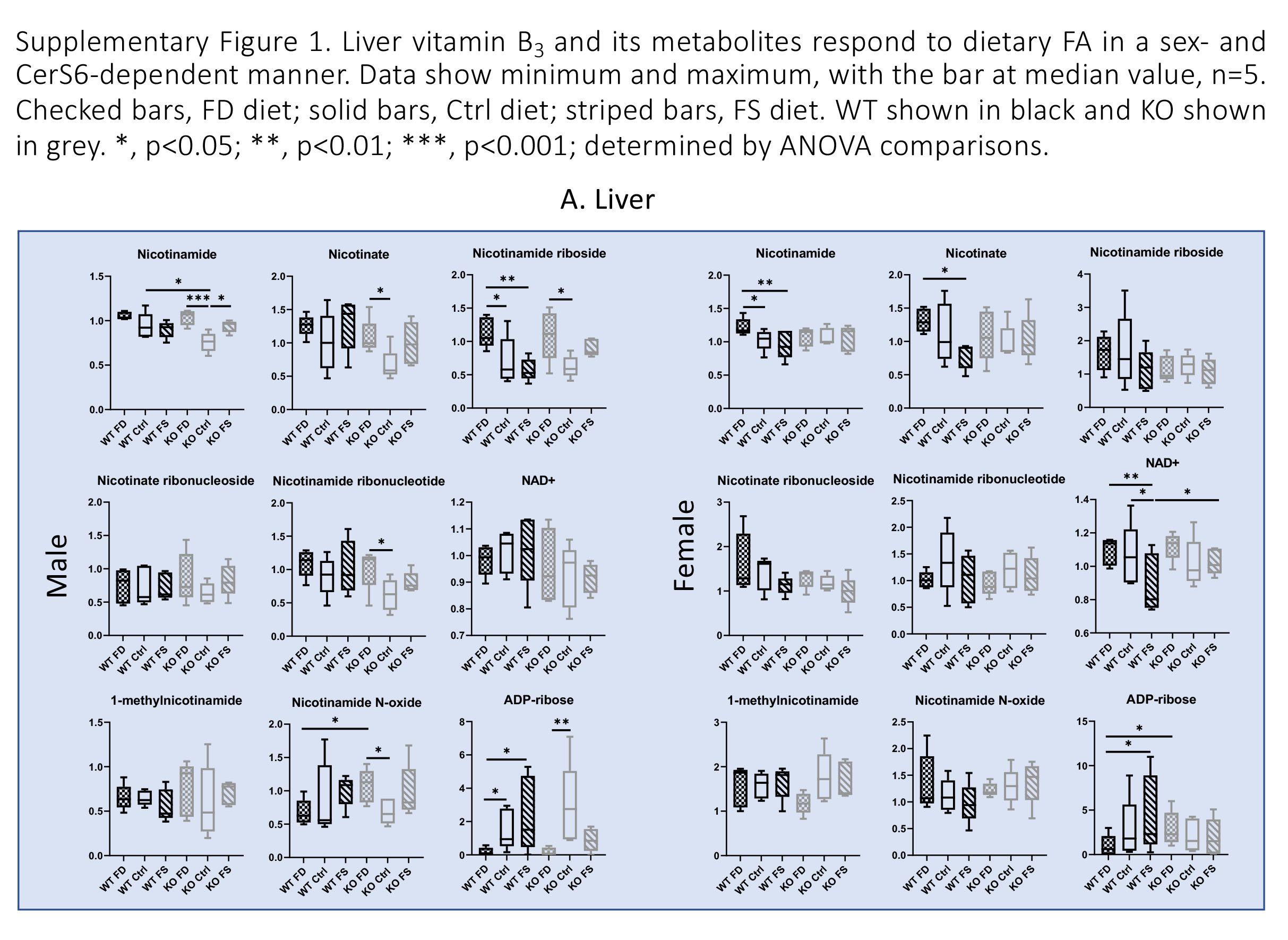

Supplement: Supplementary file 1 [file Image_1.jpg]

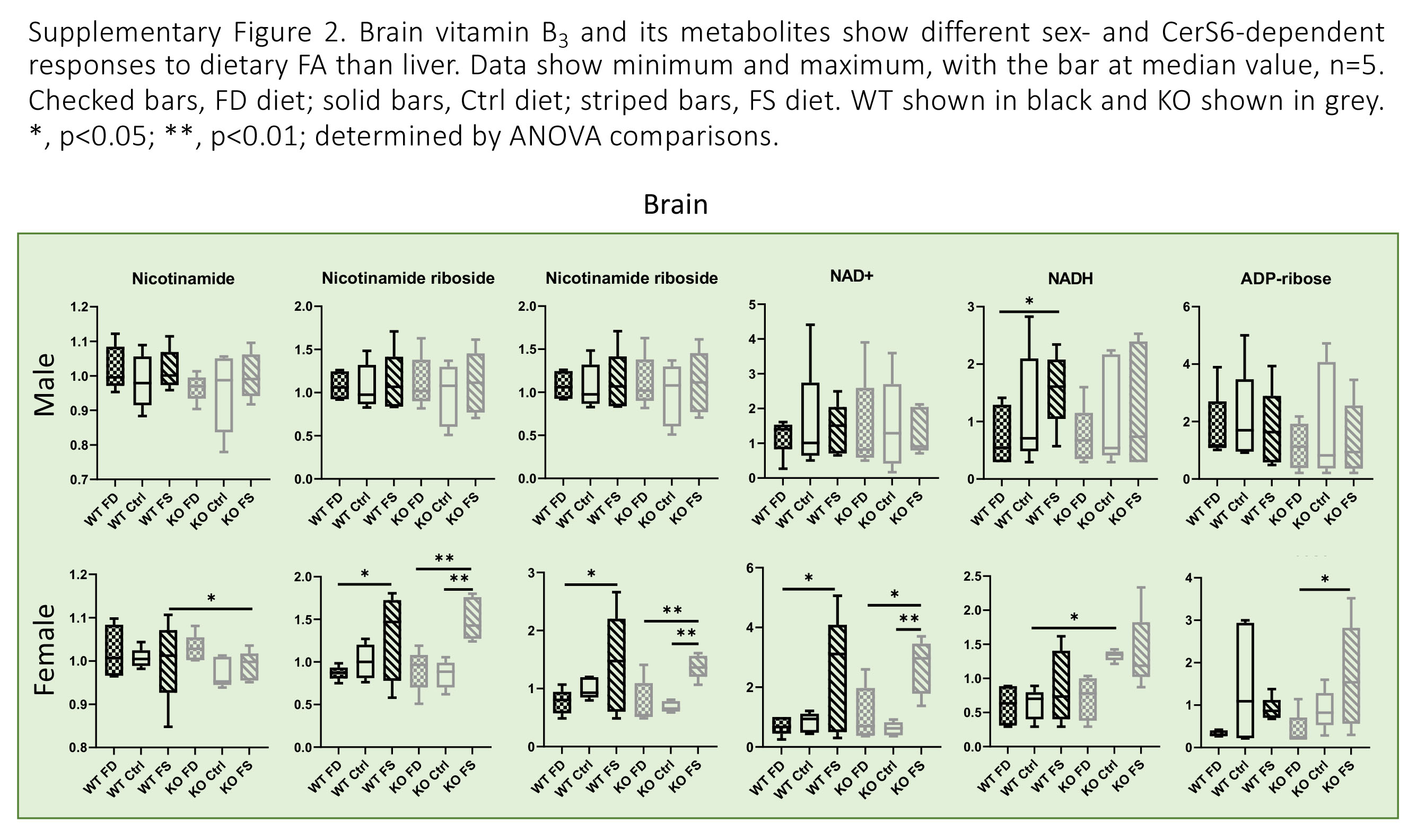

Supplement: Supplementary file 2 [file Image_2.jpg]

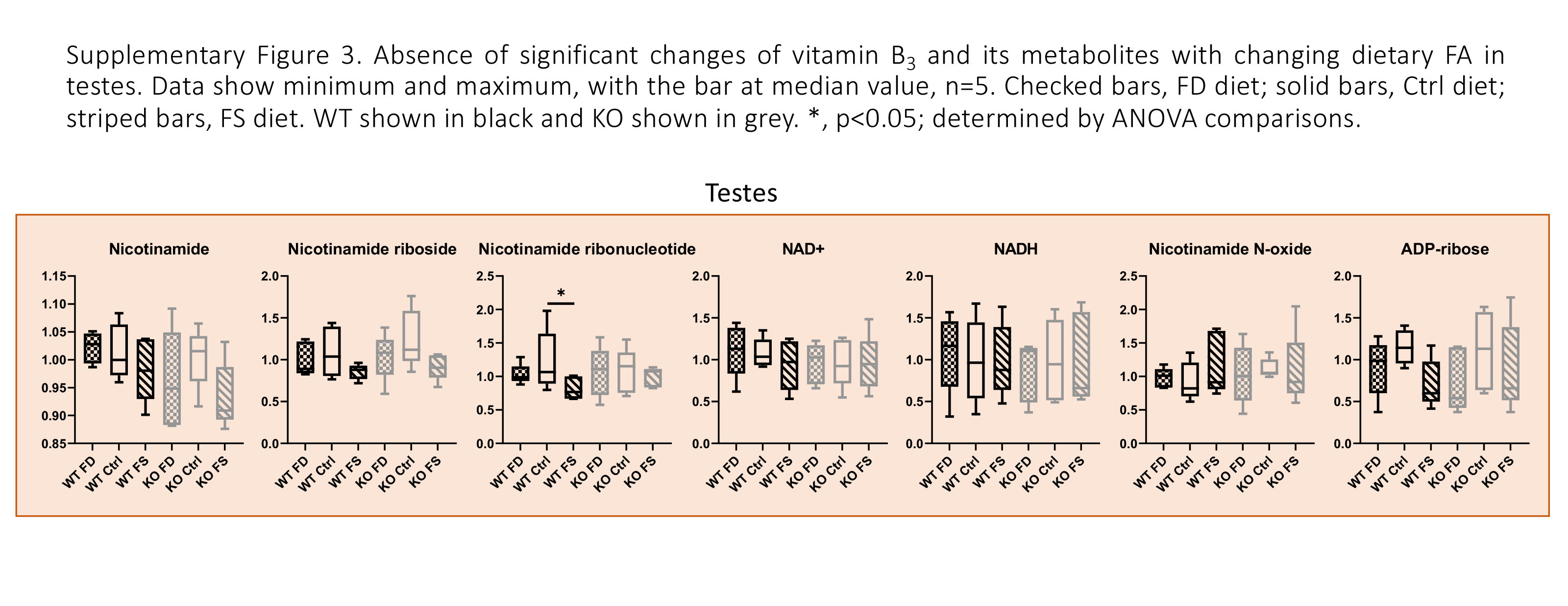

Supplement: Supplementary file 3 [file Image_3.jpg]

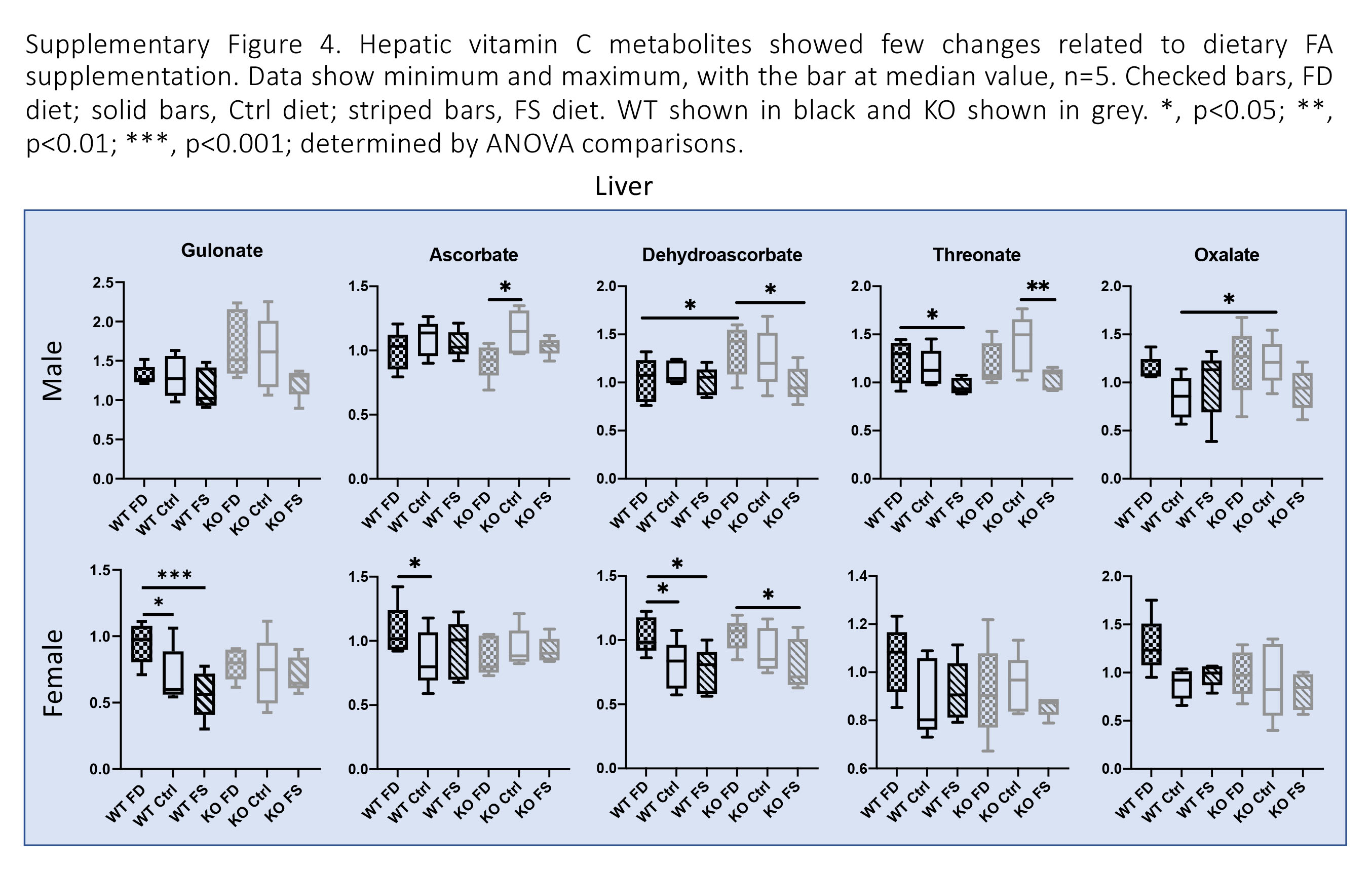

Supplement: Supplementary file 4 [file Image_4.jpg]

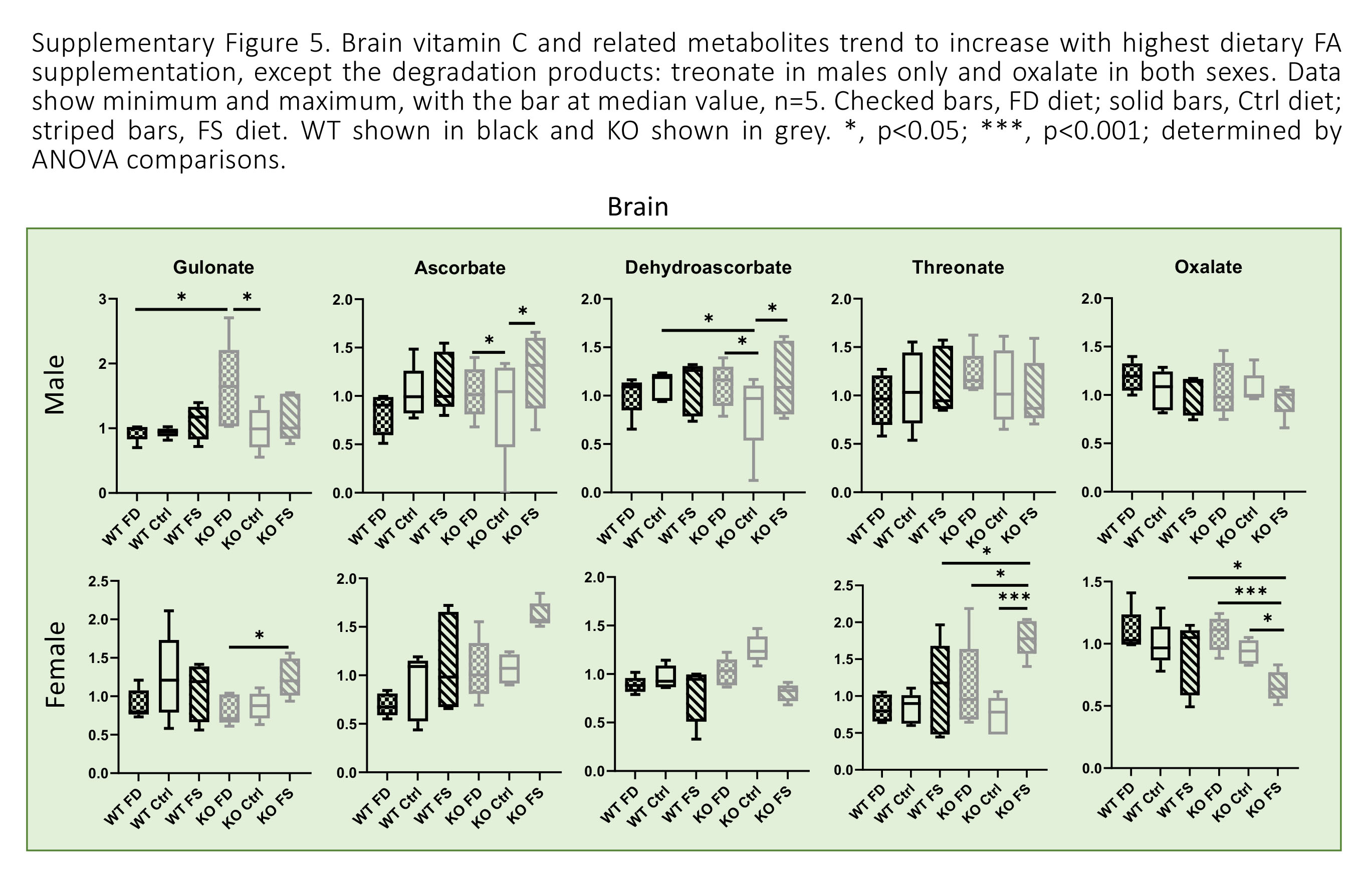

Supplement: Supplementary file 5 [file Image_5.jpg]

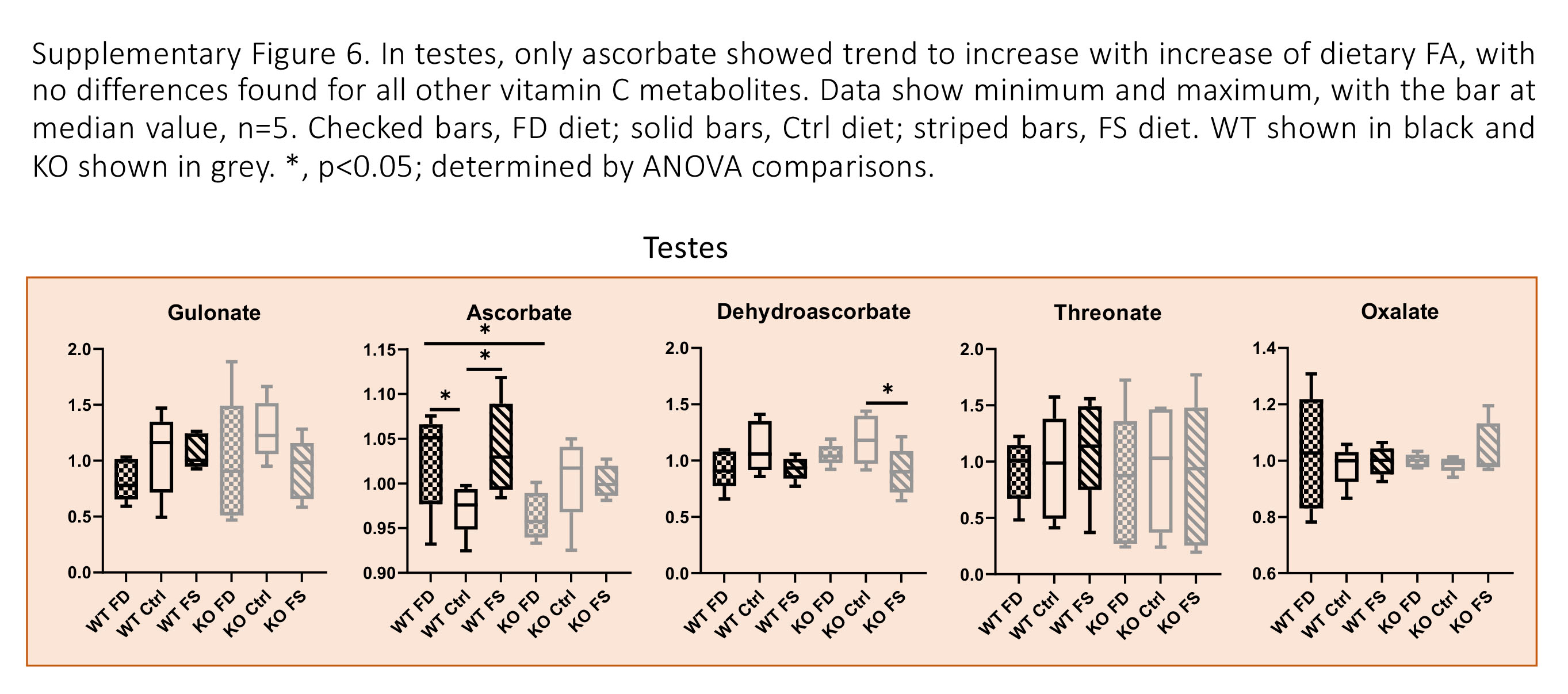

Supplement: Supplementary file 6 [file Image_6.jpg]
