# Supplementary material for: Dietary Folic Acid Alters Metabolism of Multiple Vitamins in a CerS6- and Sex-Dependent Manner
Source: Front Nutr. 2021 Nov 5;8:758403. doi: 10.3389/fnut.2021.758403 (PMC8602897; doi:10.3389/fnut.2021.758403)
Supplement: Supplementary file 7 [file Table_1.pdf]

Supplementary Table 1

Diet number/(Teklad)	*TD.95247*	*TD.160824*	*TD.160825*
Diet Name	*Folic Acid Deficient (FD)*	*Control (Ctrl)*	*Folic Acid Supplemented (FS)*
Ingredient	***g/kg***		
Caseine, Vitamin-Free	195.0	195.0	195.0
L-Cystine	3.0	3.0	3.0
Corn Starch	314.488	314.488	314.488
Sucrose	209.749	199.9	199.89
Maltodextrin	130.0	130.0	130.0
Cellulose	50.0	50.0	50.0
Soybean Oil	60.0	60.0	60.0
Mineral Mix (AIN-93G-MX)	35.0	35.0	35.0
Vitamin Mix (AIN-93-VX)	**0.0**	***10.0***	***10.0***
Folic Acid	0.0	0.002*	0.01 + 0.002*
Choline Bitartrate	2.5	2.5	2.5
TBHQ, antioxidant	0.012	0.012	0.012
Niacin	0.03	0.03*	0.03*
Calcium Pantothenate	0.016	0.016*	0.016*
Pyridoxine HCl	0.007	0.007*	0.007*
Thiamin (81%)	0.006	0.006*	0.006*
Riboflavin	0.006	0.006*	0.006*
Biotin	0.0002	0.0002*	0.0002*
Vitamin B12 (0.1% in mannitol)	0.025	0.025*	0.025*
Vitamin E, DL-alpha tocopheryl acetate (500 IU/g)	0.15	0.15*	0.15*
Vitamin A Palmitate (500,000 IU/g)	0.008	0.008*	0.008*
Vitamin D3, cholecalciferol (500,000 IU/g)	0.002	0.002*	0.002*
Vitamin K1, phylloquinone	0.0008	0.0008*	0.0008*
**% kcal/kJ from protein**	**19.0**	**19.0**	**19.0**
**% kcal/kJ from carbohydrate**	**66.6**	**66.6**	**66.6**
**% kcal/kJ from fat**	**14.4**	**14.4**	**14.4**
**kcal/g**	**3.8**	**3.8**	**3.8**
**kJ/g**	**15.899**	**15.899**	**15.899**

** Amounts added from AIN-93-VX (10 g/kg)*
