# Supplementary material for: Dietary Folic Acid Alters Metabolism of Multiple Vitamins in a CerS6- and Sex-Dependent Manner
Source: Front Nutr. 2021 Nov 5;8:758403. doi: 10.3389/fnut.2021.758403 (PMC8602897; doi:10.3389/fnut.2021.758403)
Supplement: Supplementary file 9 [file Data_Sheet_1.pdf]

Supplementary Materials – Statistical analysis performed by Metabolon®

Standard statistical analyses were performed in ArrayStudio on log transformed data. For those analyses not standard in ArrayStudio, the programs R (http://cran.r-project.org/) or JMP were used.

1. One-way ANOVA

For ANOVA, it is assumed that all populations have the same variances. One-way ANOVA was used to test whether at least two unknown means are all equal or whether at least one pair of means is different.

Often within ANOVA, one performs linear contrasts for specific comparisons of interest. For example, suppose we have three groups A, B, C, then examples of some contrasts are A vs. B, the average of A and B vs. C, etc. For single-degree of freedom contrasts, these give the same result as a two-sided *t*-test with the pooled estimate of the variance from the ANOVA and degrees of freedom $n - g$. Below, we show the three formulas for A vs. B from a three-group design as shown above. The numerator is same in each case, but the denominator differs by the estimates of the variances, and the degrees of freedom are different for each (if the theoretical assumptions hold, then the contrast has the most power, as it has the largest degrees of freedom).

Welch's two-sample *t*-test

By $t = (\bar{x}_A - \bar{x}_B)/\sqrt{s_A^2/n_A + s_B^2/n_B}$, and the degrees of freedom is given by $\left(\frac{s_A^2}{n_A} + \frac{s_B^2}{n_B}\right)^2 / \left(\frac{\left(\frac{s_A^2}{n_A}\right)^2}{n_A - 1} + \frac{\left(\frac{s_B^2}{n_B}\right)^2}{n_B - 1}\right)$

Two-sample *t*-test with pooled estimate of variance from A and B

$$t = (\bar{x}_A - \bar{x}_B)/\sqrt{s_{AB}^2(1/n_A + /n_B)}$$

where $s_{AB}^2 = \left((n_A - 1)s_A^2 + (n_B - 1)s_B^2\right)/(n_A + n_B - 2)$, where the degrees of freedom is $n_A + n_B - 2$.

The contrast from the ANOVA,

$$t = (\bar{x}_A - \bar{x}_B)/\sqrt{s^2(1/n_A + /n_B)}$$

where $s^2 = \left((n_A - 1)s_A^2 + (n_B - 1)s_B^2 + (n_C - 1)s_C^2\right)/(n_A + n_B + n_C - 3)$, where the degrees of freedom is given by where the degrees of freedom is $n_A + n_B + n_C - 3$.

2. Two-way ANOVA

For ANOVA, it is assumed that all populations have the same variances. For a two-way ANOVA, three statistical tests are typically performed: the main effect of each factor and the interaction. Suppose we have two factors A and B, where A represent the genotype and B represent the diet

in a mouse study. Suppose each of these factors has two levels (A: wild type, knock out; B: standard diet, high fat diet). For this example, there are 4 combinations ("treatments"): A1B1, A1B2, A2B1, A2B2. The overall ANOVA F-test gives the p-value for testing whether all four of these means are equal or whether at least one pair is different. However, we are also interested in the effect of the genotype and diet. A main effect is a contrast that tests one factor across the levels of the other factor. Hence the A main effect compares (A1B1 + A1B2)/2 vs. (A2B1 + A2B2)/2, and the B-main effect compares (A1B1 + A2 A2B2)/2 vs. (A1B2 + A2B2)/2. The interaction is a contrast that tests whether the mean difference for one factor depends on the level of the other factor, which is (A1B2 + A2B1)/2 vs. (A1B1 + A2B2)/2.

Some sample plots follow. For the first plot, there is a B main effect, but no A main effect and no interaction, as the effect of B does not depend on the level of A. For the second plot, notice how the mean difference for B is the same at each level of A and the difference in A is the same for each level of B, hence there is no statistical interaction. The final plot also has main effects for A and B, but here also has an interaction: we see the effect of B depends on the level of A (0 for A1 but 2 for A2), i.e., the effect of the diet depends on the genotype. We also see here the interpretation of the main effects depends on whether there is an interaction or not.

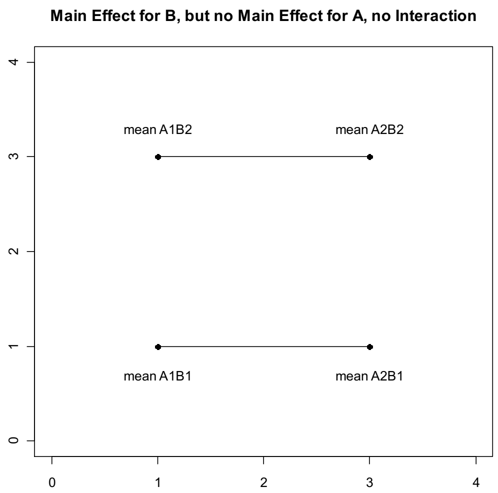

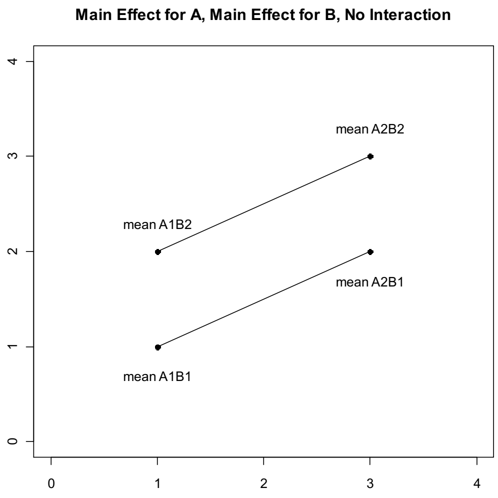

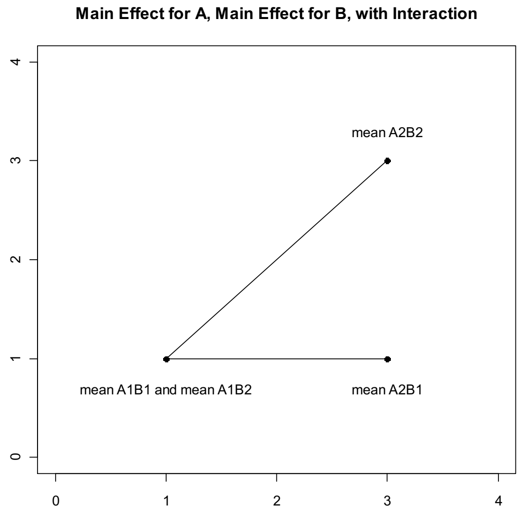

3. p- values

For statistical significance testing, p-values are given. The lower the p-value, the more evidence we have that the null hypothesis (typically that two population means are equal) is not true. If "statistical significance" is declared for p-values less than 0.05, then 5% of the time we incorrectly conclude the means are different, when actually they are the same.

The p-value is the probability that the test statistic is at least as extreme as observed in this experiment given that the null hypothesis is true. Hence, the more extreme the statistic, the lower the p-value and the more evidence the data gives against the null hypothesis.

4. q-values

The level of 0.05 is the false positive rate when there is one test. However, for a large number of tests we need to account for false positives. There are different methods to correct for multiple testing. The oldest methods are family-wise error rate adjustments (Bonferroni, Tukey, etc.), but these tend to be extremely conservative for a very large number of tests. With gene arrays, using the False Discovery Rate (FDR) is more common. The family-wise error rate adjustments give one a high degree of confidence that there are zero false discoveries. However, with FDR methods, one can allow for a small number of false discoveries. The FDR for a given set of compounds can be estimated using the q-value (see Storey J and Tibshirani R. (2003) Statistical significance for genome-wide studies. Proc. Natl. Acad. Sci. USA 100: 9440-9445; PMID: 12883005).

In order to interpret the q-value, the data must first be sorted by the p-value then choose the cutoff for significance (typically $p<0.05$). The q-value gives the false discovery rate for the selected list (i.e., an estimate of the proportion of false discoveries for the list of compounds whose p-value is below the cutoff for significance). For Table 1 below, if the whole list is declared significant, then the false discovery rate is approximately 10%. If everything from Compound 079 and above is declared significant, then the false discovery rate is approximately 2.5%.

Table 1: Example of q-value interpretation

Compound	*p*-value	*q*-value
Compound 103	0.0002	0.0122
Compound 212	0.0004	0.0122
Compound 076	0.0004	0.0122
Compound 002	0.0005	0.0122
Compound 168	0.0006	0.0122
Compound 079	0.0016	0.0258
Compound 113	0.0052	0.0631
Compound 050	0.0053	0.0631
Compound 098	0.0061	0.0647
Compound 267	0.0098	0.0939
